# ERp57 chaperon protein protects neuronal cells from Aβ‐induced toxicity

**DOI:** 10.1111/jnc.15655

**Published:** 2022-07-08

**Authors:** Daniel Di Risola, Daniela Ricci, Ilaria Marrocco, Flavia Giamogante, Maddalena Grieco, Antonio Francioso, Aldrin Vasco‐Vidal, Patrizia Mancini, Gianni Colotti, Luciana Mosca, Fabio Altieri

**Affiliations:** ^1^ Department of Biochemical Sciences Sapienza University of Roma Rome Italy; ^2^ Immunobiology of Infection Unit, Institut Pasteur Paris France; ^3^ Department of Biological Regulation Weizmann Institute of Science Rehovot Israel; ^4^ Institute of Oncology Research (IOR), Bellinzona Switzerland; ^5^ Leibniz Institute of Plant Biochemistry Halle (Saale) Germany; ^6^ Department of Experimental Medicine Sapienza University of Roma Rome Italy; ^7^ Institute of Molecular Biology and Pathology—Italian National Research Council Rome Italy

**Keywords:** Alzheimer's disease, Amyloid beta_25−35_, ER stress, ERp57, PDIA3

## Abstract

Alzheimer's disease (AD) is a neurodegenerative disorder whose main pathological hallmark is the accumulation of Amyloid‐β peptide (Aβ) in the form of senile plaques. Aβ can cause neurodegeneration and disrupt cognitive functions by several mechanisms, including oxidative stress. ERp57 is a protein disulfide isomerase involved in the cellular stress response and known to be present in the cerebrospinal fluid of normal individuals as a complex with Aβ peptides, suggesting that it may be a carrier protein which prevents aggregation of Aβ. Although several studies show ERp57 involvement in neurodegenerative diseases, no clear mechanism of action has been identified thus far. In this work, we gain insights into the interaction of Aβ with ERp57, with a special focus on the contribution of ERp57 to the defense system of the cell. Here, we show that recombinant ERp57 directly interacts with the Aβ_25−35_ fragment in vitro with high affinity via two in silico‐predicted main sites of interaction. Furthermore, we used human neuroblastoma cells to show that short‐term Aβ_25−35_ treatment induces ERp57 decrease in intracellular protein levels, different intracellular localization, and ERp57 secretion in the cultured medium. Finally, we demonstrate that recombinant ERp57 counteracts the toxic effects of Aβ_25−35_ and restores cellular viability, by preventing Aβ_25−35_ aggregation. Overall, the present study shows that extracellular ERp57 can exert a protective effect from Aβ toxicity and highlights it as a possible therapeutic tool in the treatment of AD.

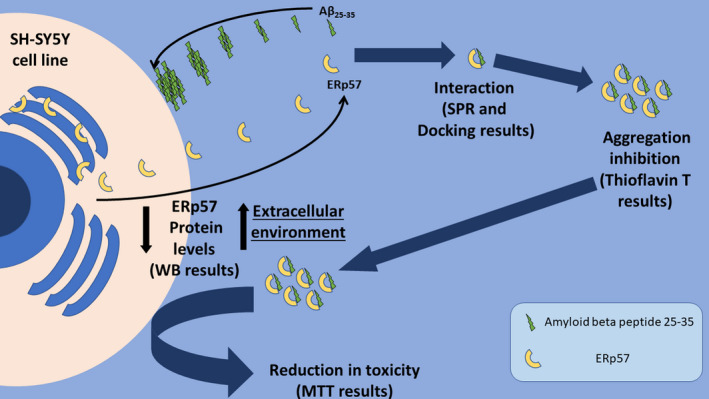

## Graphical abstract

Alzheimer's disease (AD) is a neurodegenerative disorder whose main pathological hallmark is the accumulation of Amyloid‐β peptide (Aβ) in the form of senile plaques. ERp57 is a protein disulfide isomerase involved in cellular stress response, found in the cerebrospinal fluid of healthy individuals as a complex with Aβ. Here we demonstrate that recombinant ERp57 directly interacts with the Aβ_25−35_ fragment in vitro with high affinity, leading to a reduction in Aβ aggregation and toxicity, as assessed in a cellular model of the disease. Overall, the present study shows that extracellular ERp57 can exert a protective effect from Aβ toxicity and highlights it as a possible therapeutic tool in the treatment of AD.

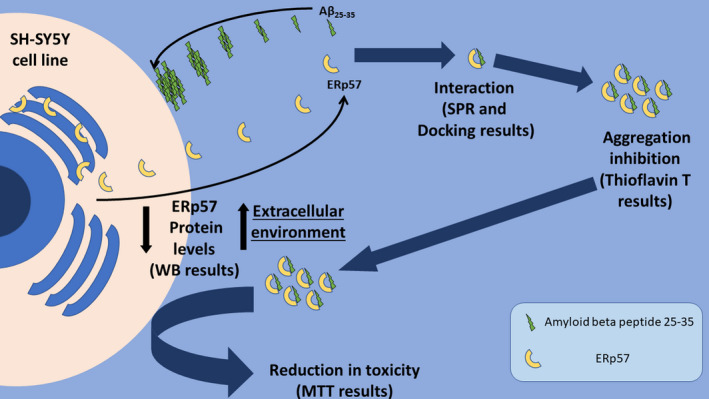

**Abbreviations**
Aβ: amyloid beta peptide
AD: Alzheimer disease
ALS: amyotrophic lateral sclerosis
APP: amyloid precursor protein
BSA: bovine serum albumin
EDTA: ethylenediaminetetraacetic acid
ER: endoplasmic Reticulum
ERAD: ER‐associated degradation process
GST: glutathione‐S‐transferase
HEPES: 4‐(2‐hydroxyethyl)‐1‐piperazineethanesulfonic acid
IPTG: isopropyl‐β‐D‐thiogalactopyranoside
PD: Parkinson's disease
PDI: protein disulfide isomerase
PMSF: phenylmethylsulfonyl fluoride
SOD‐1: super oxide dismutase‐1
SPR: surface plasmon resonance
ThT: thioflavin‐T
UPR: unfolded protein response

## 1 INTRODUCTION

Alzheimer's disease (AD) is a chronic disease that is estimated to affect about 47 million people worldwide (Tiwari et al., 2019). AD is a late‐onset disease (80–90 years of age) and is the leading cause of dementia, beginning with memory loss. In the past years, the diagnosis of genetic forms of AD has been greatly improved, but the etiology of the sporadic forms remains debated (Armstrong, 2013). Even if symptoms can be ameliorated, to date there is no treatment that can change the outcome of the disease (Sheppard & Coleman, 2020; Tiwari et al., 2019). AD is characterized by neuritic extracellular amyloid plaques in the brain, with loss of neurons and synapses (Cabral‐Miranda & Hetz, 2018; Masters et al., 2015). Neuronal damage in AD is induced by the aberrant accumulation of amyloid aggregates and neurofibrillary tangles, which consist of amyloid beta peptide (Aβ) and phosphorylated tau protein, respectively (Tiwari et al., 2019). Aβ is generated from the Amyloid Precursor Protein (APP) by the sequential action of two secretases, that is, the β‐ and γ‐secretase (Sheppard & Coleman, 2020) and many studies show that its accumulation and a conformational change into forms with a high β‐sheet structure is key in the pathogenesis of AD (Zhao et al., 2012; Zou et al., 2019). Even though the predominant forms of Aβ in the human brain are Aβ_1−40_ and Aβ_1−42_, other Aβ peptides can be present. Among them, the Aβ_25−35_ fragment can be found in the senile plaques (Kaminsky et al., 2010). Aβ_25−35_ is produced in the brain of the elderly because of the cleavage of racemized Aβ_1−40_. It has been hypothesized that Aβ_25−35_ represents the biologically active region of Aβ, being the shortest fragment retaining the toxicity of the full‐length peptide and which tends to aggregate and form fibrils (Clementi et al., 2005; Frozza et al., 2009; Millucci et al., 2010). Administration of Aβ_25−35_ recapitulates the pathological features of AD neurodegeneration and has been shown to lead to amnesia and memory loss in rats (Stepanichev et al., 2003) and to oxidative stress in the hippocampus of mice (Lu et al., 2009), suggesting its involvement in the pathogenesis of AD and its potential mechanism of toxicity. Because of this evidence, Aβ_25−35_ has often been chosen as a model in structural and functional studies of Aβ‐induced pathogenesis.

ERp57, also known as PDIA3, is a thiol oxidoreductase with Protein Disulfide Isomerase (PDI) activity. The main function of ERp57 is to promote the proper folding and to carry out quality control of newly synthesized glycoproteins in the lumen of the Endoplasmic Reticulum (ER). This enzyme works in conjunction with the lectin chaperones Calnexin and Calreticulin to ensure that secretory and membrane proteins are in the correct conformation before leaving the ER (Leach et al., 2002; Williams, 2006). ERp57 consists of four thioredoxin‐like domains, named a, b, bʹ, and aʹ. The a and aʹ domains are catalytically active, with Cys‐Gly‐His‐Cys active site motifs, and bind unfolded proteins substrates, while the b and bʹ domains contain a positively charged calnexin binding site, that assists in the substrate recruitment process (Dong et al., 2009a; Kozlov et al., 2006). Activities of ERp57 were reported in different cellular compartments (Turano et al., 2011). It binds STAT3 in the cytoplasm and ER (Coe et al., 2010), in the nucleus it has DNA binding ability (Chichiarelli et al., 2007; Coppari et al., 2002), and it has been identified on the plasma membrane as the cell surface receptor for the metabolite 1,25‐dihydroxycholecalciferol (Boyan et al., 2012; Nemere et al., 2004). Intriguingly, ERp57 has been detected in the extracellular space of different cell types, for example, to suppress complement activation or as an early sign of renal fibrosis onset (Dihazi et al., 2013; Eriksson et al., 2019), suggesting that ERp57 might have different, currently unknown, extracellular functions. Moreover, ERp57 is a stress‐responsive protein which is up‐regulated in response to ER stress (Nundlall et al., 2010; Turano et al., 2011). ER stress happens when the equilibrium between folding capacity and protein load in the cell is altered and misfolded proteins accumulate in the ER. As a result, the cell initiates a signaling network called Unfolded Protein Response (UPR) in an attempt to reestablish the cellular homeostasis (Ghemrawi & Khair, 2020). Precisely, ERp57 is one of the players that enhance the folding capacity of the cell (Bargsted et al., 2016). Chronic ER stress has been linked to different neurodegenerative diseases, which share the common feature of accumulation of protein aggregates in the central nervous system, like AD, Parkinson's disease (PD), Huntington disease, and Amyotrophic Lateral Sclerosis (ALS). It has been proven that the expression of the misfolded protein characteristic of the disease is sufficient to induce UPR in cellular and animal models (Cabral‐Miranda & Hetz, 2018; Gerakis & Hetz, 2018).

ERp57 has been previously linked to key processes in neurodegeneration and particularly in AD pathogenesis. It has been demonstrated that cerebrospinal fluid of healthy individuals contains Aβ peptide complexed with ERp57 and calreticulin(Erickson et al., 2005) and that ERp57 interacts with full‐length APP in the early secretory pathway of this protein (Selivanova et al., 2007). This evidence suggests that ERp57 interacts with Aβ and may play a role in preventing its aggregation. As evidence of this, activation of ERp57 with diosgenin significantly improves performance of object recognition memory and reduces amyloid plaques and neurofibrillary tangles in the cerebral cortex and hippocampus in the AD 5XFAD mice model (Tohda et al., 2012). Moreover, involvement of ERp57 in other neurodegenerative diseases has been demonstrated. Indeed, it was found that the expression and function of ERp57 could be modulated under conditions of oxidative stress in neuronal cells in culture and in a PD animal model (Aureli et al., 2014; Giamogante et al., 2018), suggesting a feedback loop mechanism, and that ERp57 has a protective role to motor function in early stages of ALS progression, preserving the neuromuscular junction (Rozas et al., 2021) and in prion neurotoxicity (Hetz et al., 2005; Thapa et al., 2018).

In this work, we show how ERp57 is involved in the cellular response to Aβ‐induced stress through a previously unreported mechanism. In this respect, we employed SH‐SY5Y neuroblastoma cells treated with micromolar amounts of Aβ_25−35_, a well‐established AD cellular model (Xicoy et al., 2017), to investigate the effects of aggregated Aβ_25−35_ on ERp57 protein levels and localization. Interestingly, Aβ_25−35_ treatment reduced the intracellular protein levels of ERp57 while, in parallel, a significant increase in extracellular ERp57 was reported. This evidence was also supported by ERp57 intracellular relocalization upon Aβ_25−35_ stimulation. Finally, we reported the binding between ERp57 and Aβ_25−35_ in vitro and examined whether a direct interaction between Aβ_25−35_ and ERp57 in vitro leads to a reduction in cell mortality, outlining a possible buffering effect of ERp57 on Aβ_25−35_‐induced toxicity. Altogether, our results prompt us to hypothesize that ERp57 is secreted in the extracellular space in response to Aβ_25−35_ insult to bind and counteract the toxic effects of the aggregated Aβ_25−35_.

## 2 METHODS

### 2.1 Chemicals

Unless otherwise stated, reagents were purchased from Sigma Aldrich. Rabbit polyclonal anti‐ERp57 antibody against recombinant human ERp57 was generated and purified in our lab by affinity chromatography (Coppari et al., 2002); secondary antibodies goat polyclonal anti‐rabbit IgG (Alexa Fluor® 488) were from Abcam (catalog number #ab150077); culture medium (Catalog number #21331020) and serum (Catalog number #10270106) were from Gibco BRL (Life Technologies Inc.); goat Anti‐Rabbit (Catalog number #1706515) and Anti‐mouse (Catalog number #1706516) IgG (H + L)‐HRP Conjugate, Bradford (catalog number #5000205) reagent and Criterion TGX stain‐free 4–20% acrylamide gel (Catalog number #4568093) were purchased from Biorad®.

### 2.2 Preparation of Aβ_25−35_ stock and working solution

The amyloid peptide Aβ_25−35_ was synthesized by conventional solid‐phase chemistry (Atherton & Sheppard, 1990), and stored at −20°C. The peptide was resuspended in 1,1,1,3,3,3‐Hexafluoro‐2‐propanol (Sigma®, catalog number #105228), incubated for 1 h with gentle shaking at 4°C, then aliquoted and dried in speedVac for 20 min. The aliquots were stored under a vacuum glass bell. The day before being used, aliquots were resuspended in phosphate‐buffered saline (PBS) at a final concentration of 1 mM, incubated in a ultrasonic bath on ice for 30 min to induce aggregation, followed by gentle shaking at 4°C overnight. Aβ_25−35_ was diluted in culture medium at a final concentration of 30 μM for cell treatments. Aggregation of Aβ_25−35_ in the presence of ERp57 was obtained as follows: dried aliquots of Aβ_25−35_ were resuspended in PBS. Solutions were sonicated in ultrasonic bath for 30 min in ice, purified ERp57 or GST proteins were added to the solution to reach the final concentration of 4 μM for ERp57/GST and 1 mM for Aβ_25−35_ (in a ratio 1:250, defined by preliminary experiments of cell viability) and incubated for 72 h under gentle shaking at 4°C. Every 24 h an aliquot of 10 μl was collected from each sample and tested in ThT assay.

### 2.3 ERp57 protein production

Human recombinant PDIA3 was cloned and expressed in *E. coli* strain BL21 (Invitrogen™, catalog number C600003) using the expression vector pET21 (Novagen, catalog number 69770) as described by Coppari (Coppari et al., 2002). The coding sequence for the second redox‐active domain (aʹ domain, residues 377–505) was amplified by PCR as previously described and cloned in the expression vector pET29 (Novagen) (Grillo et al., 2002). Recombinant proteins were expressed in *E. coli* strain BL21 and purified by ammonium sulfate fractionation, ion exchange, and heparin chromatography (Grillo et al., 2006). Protein purification was evaluated by SDS‐PAGE and concentration was determined spectrophotometrically (PDIA3 Ɛ_280_ reduced form = 44 810 M^−1^ cm^−1^, aʹ domain Ɛ_280_ reduced form = 14 400 M^−1^ cm^−1^). Rabbit anti‐ERp57 primary antibody will be shared upon reasonable request.

### 2.4 GST protein production

To express GST, the plasmid pGEX‐4 T‐1 (catalog number #28954549, GE healthcare), bearing the GST gene, was transformed into the *E. coli* BL21 (DE3) strain. The recombinant bacteria were grown in 1‐liter LB medium (catalog number #L3397) in the presence of ampicillin (50 μg/ml) (catalog number #A9393). When the culture had reached an optical density (OD600) of 0.6, isopropyl‐β‐D‐thiogalactopyranoside (IPTG) (catalog number #PHG0010) 1 mM was added to induce protein expression and then growth was continued for 3 h. The cells were spun down at 3000 g, resuspended in 50 mM HEPES +150 mM NaCl +0.1 mM EDTA at pH 7.4 (buffer A) + 1 mM PMSF (catalog numbers #H3375, #S9888, #E9884, #PMSF‐RO), sonicated and centrifuged at 15 000 *g* for 45 min. The supernatant was loaded onto a GSTrap FF affinity column (catalog number #11340212 GE healthcare), equilibrated with buffer A, washed extensively with buffer A, and eluted with elution buffer (50 mM Tris–HCl, 10 mM reduced glutathione, pH 8.0) (catalog numbers #10812846001, #G4251) at 1 ml/min flow rate. Optical density was monitored at 280 nm using the optical unit of a liquid chromatography system (AKTA P‐900, GE Healthcare BioScience AB). A putative peak containing the recombinant GST protein was identified at 20% elution buffer, collected for analysis, and dialyzed in 20 mM HEPES at pH 7.5. The total protein concentration of each fraction was determined using a Micro BCA kit (catalog number #23252, Pierce) using bovine serum albumin as the reference protein. The purity of the protein sample was analyzed using aliquots of the fractions by 12% SDS‐PAGE (catalog number #5678044, Biorad) and western blotting with appropriate antibodies.

### 2.5 Cell culture and treatment

SH‐SY5Y (RRID:CVCL_0019) cells were obtained from the ICLC. The cell line is not listed as a commonly misidentified cell line by the ICLAC (http://iclac.org/databases/cross‐contaminations/). Therefore, we did not perform any authentications of these cell lines.

Cells were grown in DMEM/F‐12 medium (Catalog number #21331020) containing 10% fetal bovine serum (Catalog number #10270106) (Gibco BRL Life Technologies Inc.), Penicillin–Streptomycin solution (10 000 U/ml) (Catalog number #15140148) and 2 mM L‐glutamine (catalog number #25030081) at 37°C in a humidified atmosphere with 5% CO_2_. Cells were plated twice a week by trypsinization (0.5% trypsin in PBS) in 25‐cm^2^ flasks with a 4 × 10^4^ cells/cm^2^ density. Cells were used for the experiments between the 20th and 30th passage. Cells were plated at an appropriate density according to each experimental setting and treated with 30 μM aggregated Aβ_25−35_ diluted in DMEM/F12. The same volume of PBS (catalog number #P4417) was added to the control wells.

### 2.6 Immunofluorescence microscopy

Cells were seeded on 18 × 18 mm glass coverslips in 6‐well plates at a density of 5 × 10^5^ cells/well. After treatment with 30 μM Aβ_25−35_ cells were fixed with 2% (w/v) formaldehyde in PBS, washed and permeabilized with 0.1% (v/v) Triton X‐100 (catalog number #X100) in PBS. After washing with PBS, the coverslips were exposed for 30 min to blocking buffer (5% (w/v) Bovine Serum Albumin BSA (catalog number #P6154‐100GR, Biowest) in PBS and then incubated overnight at 4°C with the rabbit anti‐ERp57 antibody (1:100 dilution) in 1% (w/v) BSA in PBS. Coverslips were washed with PBS before incubation for 1 h with the secondary antibody Alexa Fluor® 488 goat anti‐rabbit IgG (1:500 dilution) in 1% (w/v) BSA in PBS. Nuclei were stained for 1 min with 1 μg/ml of 4′,6‐diamidino‐2‐phenylindole dihydrochloride (DAPI) (catalog number #D8417) in PBS and the coverslips were mounted with Mowiol (catalog number #81381). Fluorescence signals were analyzed by capturing images using an AxioObserver inverted microscope, equipped with the ApoTome System (Carl Zeiss Inc.). Microscopy imaging was performed using the Axiovision software (Zeiss).

### 2.7 MTT assay

For the cell viability experiments, 96‐well plates were seeded with 10 000 cells/well. After 24 h, cells were treated with 30 μM Aβ_25−35_ aggregated for 72 h in the presence or in the absence of 0.12 μM of ERp57 or GST recombinant proteins (in a 1:250 ratio), in a final volume of 150 μl per well. Plates were incubated for 24 h in an incubator at 37°C, 5% of CO_2_, and 99% RH. Thereafter, 20 μl of (3‐[4,5‐Dimethylthiazol‐2‐yl]‐2,5‐Diphenyltetrazolium Bromide) (MTT) dye (stock solution 5 mg/ml in PBS) (catalog number #M2003) was added to the wells and incubated for additional 2 h at 37°C and 5% CO_2_. Water‐insoluble formazan crystals were dissolved in 100 μl of DMSO (catalog number #472301). For spectrophotometric measurements, Appliscan® plate reader (Thermo scientific) was used at the wavelength 570 nm, with reference set at 690 nm.

### 2.8 Thioflavin‐T (ThT) assay

Thioflavin‐T assay was used to assess the aggregation of the Aβ_25−35_. Thioflavin‐T (catalog number #T3516) was prepared in glycine buffer (0.05 M, pH 8.5) (catalog number #G8898), to a final concentration of 5 μM, in order to be saturated by Aβ_25−35_ (10 μM). Aβ_25−35_ was aggregated either alone or in the presence of 0.04 μM ERp57 or GST recombinant protein (in a 1:250 ratio). The assay was performed using a spectrofluorometer Spex Fluoromax® at 25°C; the excitation wavelength was set at 440 nm (5 nm band width) and the fluorescence was measured between 450 and 520 nm (5 nm band width) (Francioso et al., 2020).

### 2.9 Evaluation of ERp57 protein levels by western blot

After treatment with Aβ_25−35_, SH‐SY5Y cells were lysed in RIPA buffer (50 mM Tris–HCl, pH 7.4, 150 mM NaCl, 1% NP‐40, 0.1% SDS, 1 mM EDTA, 5 mM NaF, 0.5% sodium deoxycholate) containing 1 mM PMSF, 1 mM Na_3_VO_4_ (catalog number #S6508) and SIGMAFAST™ Protease Inhibitor Cocktail (catalog number #S8820). The lysates were incubated on ice for 30 min, sonicated and centrifuged at 12 000 *g* for 20 min at 4°C. Supernatants were collected and protein quantification was performed using a Bradford Assay (BIORAD®). Equal amounts of proteins (20 μg) were separated on 4–20% SDS‐PAGE and transferred to PVDF membrane probed with the primary anti‐ERp57 antibody (1:2000), and secondary peroxidase‐conjugated antibody anti‐rabbit (1:5000) (BIORAD®). The protein bands were visualized by ECL system (catalog number #1705060, BIORAD®) according to the manufacturer's instructions. Membranes were stripped and reprobed with anti‐β‐actin monoclonal Antibody (1:5000) (Millipore) and secondary peroxidase‐conjugated antibody anti‐mouse (1:5000) (BIORAD®). Densitometric analyses were performed with ImageLab software (BioRad) (RRID:SCR_014210) and normalized to actin band. Intracellular ERp57 protein levels were expressed as a percentage compared to the control cells. In order to evaluate the release of ERp57 in culture medium, following treatments, supernatants were collected, centrifuged at 14 000 *g* for 20 min, and filtered onto 10 kDa membrane Microcon centrifugal filter (catalog number #ufc501096, Amicon). The concentrated material was lyophilized, resuspended in 100 μl of RIPA buffer and subjected to western blot as described above. Equal amounts of volume (equivalent to 30 μl of cell culture supernatants) were loaded onto the gel. Results are presented as averages of four or seven replicates for the determination of the intracellular or extracellular expression of ERp57, respectively.

### 2.10 Surface plasmon resonance

SPR experiments were performed with a SensiQ Pioneer apparatus. ERp57 immobilization was carried out essentially as previously reported (Genovese et al., 2020; Poser et al., 2017). ERp57 was immobilized via amine coupling onto a COOH5 sensorchip, previously activated by 100 μl injection of a 1:1 mixture of N‐ethyl‐N′‐3‐(diethylaminopropyl)carbodiimide (200 mM) (catalog number #39391) and N‐hydroxysuccinimide (50 mM) (catalog number #130672). Immobilizations were carried out in 20 mM sodium acetate (catalog number #236500) at pH 4.5; the unreacted groups were blocked by injecting 100 μl of 1 M ethanolamine hydrochloride (catalog number #E6133) at pH 9.5. The amount of immobilized ERp57 was detected by mass concentration‐dependent changes in the refractive index on the sensorchip surface and corresponded to about 4000 resonance units (RU). Aβ_25−35_ was diluted in sterile HEPES 20 mM, pH 7.4, NaCl 150 mM, 0.005% surfactant Tween‐20 (catalog number #P1379) (HBSP buffer) to a final concentration of 1 mM. All the experiments were carried out at 25°C in degassed HBSP buffer. Aβ_25−35_ was automatically diluted and injected for 240 s at a flow rate of 30 μl/min, at the following concentrations: 15.6 μM; 31.2 μM; 125 μM; 250 μM; 500 μM; 1.0 mM. The increase in RU relative to baseline indicates complex formation between the immobilized ERp57 ligand and the Aβ_25−35_ analyte (0–240 s). The plateau region represents the steady‐state phase of the interaction. The decrease in RU after 240 s indicates analyte dissociation from the immobilized ERp57 upon HSBP buffer injection. As a negative control, sensor chips were treated as described above in the absence of immobilized ERp57. Values of the plateau signal at steady‐state (Req) and full fittings with 1, 2, and 3 sites were calculated from kinetic evaluation of the sensorgrams using the Qdat 4.0 program.

### 2.11 Bioinformatic analysis

The FASTA sequence of the Human ERp57 protein was retrieved from the Uniprot server (UniProt) (Bateman et al., 2021).

Using the HHPRED server (HHpred | Bioinformatics Toolkit (mpg.de]) (Gabler et al., 2020), the related crystallographic structures were searched using the ERp57 sequence as input. From Protein Data Bank (PDB) (rcsb.org) (Berman et al., 2000) we selected the full‐length protein structure (pdb ID 3F8U_C) (Dong et al., 2009b) because of the low e‐value (3e^−47^) and the high quality (2.6 Å).

The docking of the most recent structure of the Aβ_25−35_ fragment (pdb: 1QXC)(D'Ursi et al., 2004) against Erp57 was performed through the HPEPDOCK server (HPEPDOCK Server (hust.edu.cn]) (Zhou et al., 2018). HPEPDOCK ranks the predicted poses using an energy‐based score, and we took in consideration the top 10 poses. Contacts, H‐bonds and clashes analysis, the hydrophobic surface analysis, and picture acquisition were performed using UCSF Chimera 1.16 software developed by the Resource for Biocomputing, Visualization, and Informatics at the University of California, San Francisco, with support from NIH P41‐GM103311 (UCSF Chimera Home Page) (Pettersen et al., 2004).

### 2.12 Statistical analysis

Experiments were repeated at least in triplicate and all the results are expressed as the mean value ± standard deviation (SD).

No formal randomization procedures were applied when allocating treatments to different experimental groups, and no blinding was performed during data analysis. No exclusion criteria were predetermined. An assessment of the normality of the data was not performed. No corrections were applied. No sample calculation was performed. No test for outliers was conducted.

Post hoc power analysis was performed using G*Power 3.1 software (Faul et al., 2007) which verified that the sample size was sufficiently powered (input parameters: two tails, 0.05 α error probability, 0.95 (1‐β error) power and effect size d was calculated using mean and standard deviation (σ values of groups).

p‐values were calculated using a two‐tailed Student's *t*‐test or one‐way or two‐way analysis of variance (ANOVA) with Tukey's multiple comparison post‐tests. *p* values <0.05 were regarded as significant. All statistical analyses were performed using Prism 6 software (GraphPad)(RRID: SCR_002798). Full statistical report is included in supplementary.

In western blot analyses, two extracellular samples (1–3 h) were excluded because it exceeded ±2 SD. One sample (24 h, intracellular and extracellular) was lost.

### 2.13 Ethics approval

No ethical approval was required for the study.

## 3 RESULTS

### 3.1 Aβ_25−35_ treatment of neuronal cells in culture decreases intracellular and increases extracellular protein levels of ERp57

**FIGURE 1:**
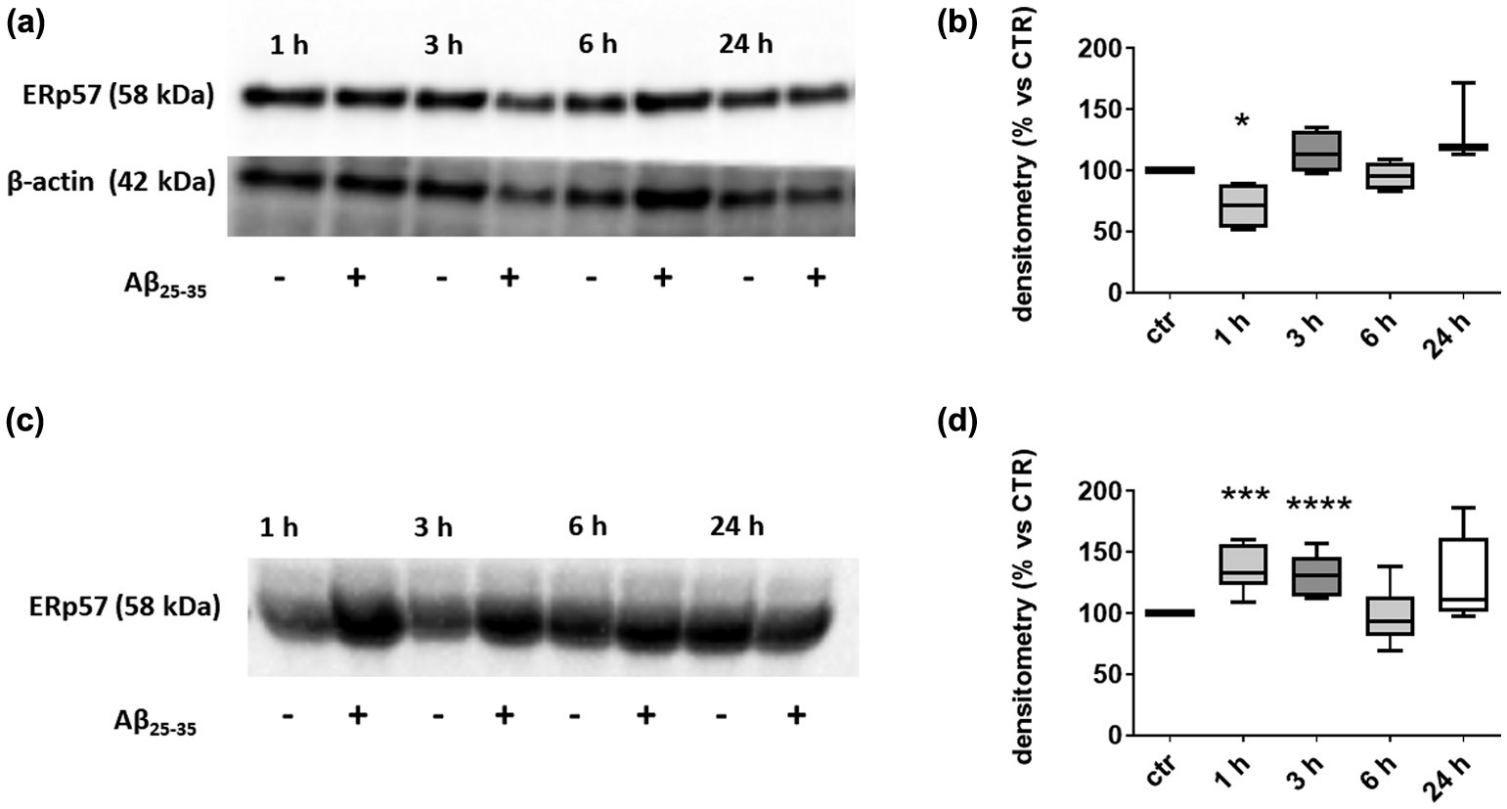
Intracellular and extracellular levels of ERp57 in SH‐SY5Y cells after treatment with Aβ_25−35_. (a) SH‐SY5Y cells were treated with 30 μM Aβ_25−35_ for the indicated time points, then cells were harvested, lysed in RIPA buffer and subjected to western blot analysis with an anti‐ERp57 antibody. β‐actin was used as a loading control. (b) Densitometric analyses were performed using ImageLab software and normalized to β‐actin. One sample (24 h) was lost. (c) Aliquots of culture medium were collected at the indicated time points and analyzed by western blot with an anti‐ERp57 antibody. Equal amounts of volume were loaded onto gels. (d) Densitometric analyses were performed with ImageLab software. ERp57 protein levels are presented as a percentage compared to the respective control at the same timepoint. Two extracellular samples (1–3 h) were excluded because it exceeded ±2 SD. One sample (24 h) was lost. Data are represented as box plot, lines represent the median and the whiskers represent min and max values (𝑛 = 4 for intracellular levels, 𝑛 = 7 for extracellular levels) 𝑛 = number of independent cell culture preparations, *p < 0.05 versus control; ***p < 0.001 versus control; ****p < 0.0001 versus control. Statistical comparison between control and treatment at each time point was performed using unpaired Student's *t*‐test.

Increasing evidence from the literature indicate that, under stress conditions, ERp57 expression can be dysregulated (Dihazi et al., 2013; Hartley et al., 2010; Nundlall et al., 2010; Piróg et al., 2019; Wang et al., 2020) and, sometimes, secreted in the extracellular space by different cell types (Dihazi et al., 2013; Hirano et al., 1995; Holbrook et al., 2012; Wang et al., 2020). With the aim to understand whether Aβ_25−35_ insult is a stress condition responsible of ERp57 protein levels alteration in neurons, we treated SH‐SY5Y neuroblastoma cells with micromolar amounts of Aβ_25−35_. Notably, treatment of SH‐SY5Y cells with 30 μM Aβ_25−35_ led to a reduction of the intracellular content of ERp57 of about 25% within the first hour of treatment (Figure 1a,b). At the subsequent time points (3–24 h), the amount of ERp57 in the cells was not significantly different compared to control cells non‐stimulated with Aβ_25−35_.

Parallel to the intracellular reduction of ERp57 levels, we observed a strong and significant release of ERp57 in the culture medium within the first 3 h of treatment (+30%) which gradually decreased in the following hours (Figure 1c,d). After 24 h, ERp57 protein levels returned to basal conditions both intracellularly and extracellularly. These data suggest that a short time stimulation with Aβ_25−35_ is sufficient to induce the reduction of intracellular ERp57 and an increase of its release in the extracellular space in a similar timeframe.

### 3.2 ERp57 cellular localization upon Aβ_25−35_ treatment

**FIGURE 2:**
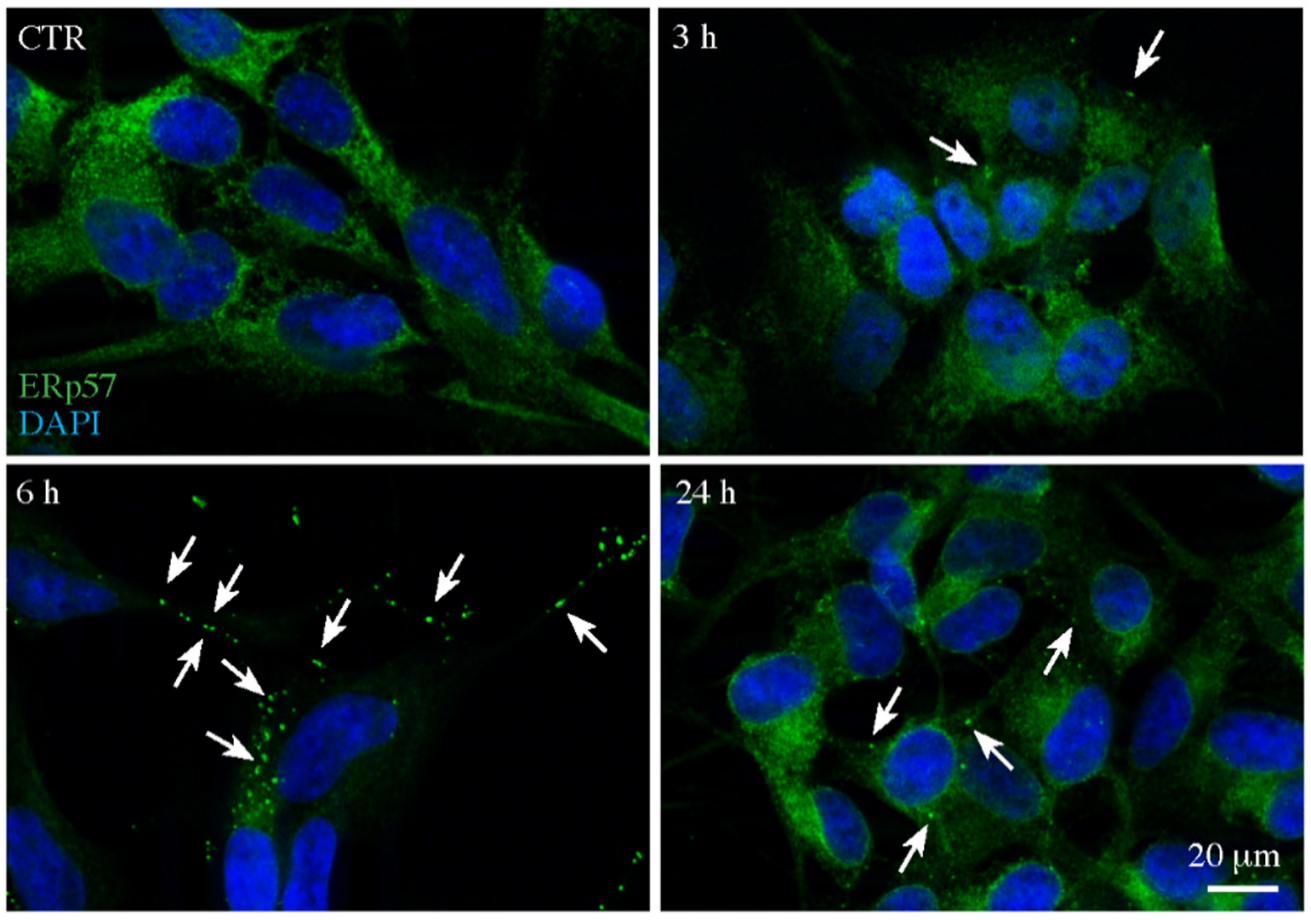
Intracellular localization of ERp57 protein after treatment with Aβ_25−35_. Representative immunofluorescence microscopy images were obtained by using an AxioObserver inverted microscope, equipped with the ApoTome system. Cells were stained with an anti‐ERp57 antibody (green) and DAPI (blue) after treatment with 30 μM Aβ_25−35_.

ERp57 is localized in different cellular compartments, though it is prevalently found in the ER where it exerts its disulfide‐isomerase and redox activities (Turano et al., 2011). Since we observed an ERp57 secretion upon Aβ_25−35_ treatment, we evaluated ERp57 intracellular localization by immunofluorescence. As expected, in control conditions, ERp57 appears as a widespread intracellular signal, mainly ascribable to the ER compartment (Figure 2). Notably, the treatment with Aβ_25−35_ caused a rearrangement of ERp57 protein localization at the cell periphery. Indeed, after 3 h of Aβ_25−35_ treatment, some fluorescence aggregates appear far from the nucleus, suggesting an early rearrangement of ERp57 protein localization. This phenomenon became more evident after 6 h, where a spotted pattern of ERp57 displays a lower perinuclear localization. This rearrangement could be attributable to the main function of ERp57, that is the correct folding of the glycosylated proteins at the level of the synapses (Holtzman, 2013). Twenty‐four hours after treatment, the cells reacquired the original redistribution, with more perinuclear signal typical of the ER, demonstrating that Aβ_25−35_ exerts its effects on ERp57 mainly after short time stimulations. These data indicate that the localization of ERp57 protein is directly affected by Aβ_25−35_ treatment within 3–6 h and suggest that an ERp57 sub‐pool gets to aggregate and move in a lower perinuclear space, a possible response to ERp57 secretion in the first 1–3 h of stimulation (Figure 1).

### 3.3 Surface plasmon resonance experiments demonstrate a direct interaction between ERp57 and Aβ_25−35_

Although evidence from the literature reports the biological link between Aβ and ERp57, data showing their direct interaction are lacking. To evaluate whether the biological effects, reported here and elsewhere, were because of direct interaction between Aβ_25−35_ and ERp57, we performed in vitro surface plasmon resonance (SPR) experiments with recombinant ERp57 and non‐aggregated Aβ_25−35_, aimed at defining whether they interact directly and the physicochemical characteristics of their interaction.

**FIGURE 3:**
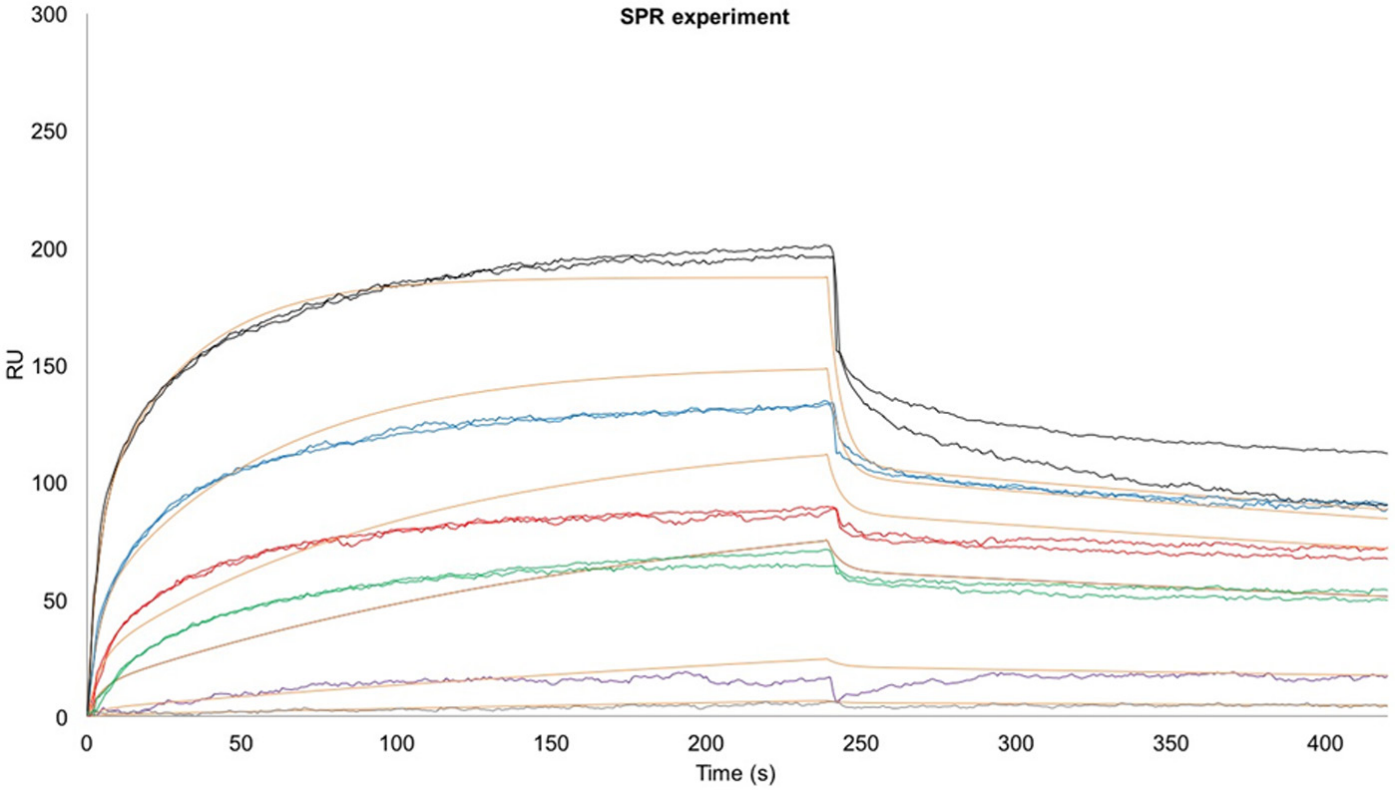
Sensorgrams showing the binding of Aβ_25−35_ to ERp57 immobilized onto a COOH5 sensorchip. Aβ_25−35_ was injected for 240 s at a flow rate of 30 μl/min, at the following concentrations: 15.6 μM (gray); 31.2 μM (purple); 125 μM (green); 250 μM (red); 500 μM (blue); 1.0 mM (black). The increase in resonance units (RU) relative to baseline indicates complex formation between the immobilized ERp57 ligand and the Aβ_25−35_ analyte (0–240 s). The plateau region represents the steady‐state phase of the interaction. The decrease in RU after 240 s indicates analyte dissociation from the immobilized ERp57 upon buffer injection. Full fittings with two binding sites calculated from kinetic evaluation of the sensorgrams using the Qdat 4.0 program (orange lines) yielded *K*_D1_ = 34 ± 2 μM, and *K*_D2_ = 3.3 ± 0.4 mM.

The ERp57 protein was immobilized on a COOH5 sensorchip, and Aβ_25−35_ was automatically injected at different concentrations. The sensorgrams obtained (Figure 3) show that ERp57 binds Aβ_25−35_ with an overall *K*_D_ of 33 ± 2 μM when global fitting with one binding site was used (data not shown). The best overall fitting was obtained by fitting the sensorgrams with two binding sites, evident in the dissociation curves, in which the presence of a “fast” and a “slow” exponential curve is clear. The fitting with two binding sites shows a *K*_D1_ = 34 ± 2 μM (very similar to the calculated overall *K*_D_), and *K*_D2_ = 3.3 ± 0.4 mM.

The dissociation phase is biphasic, an indication that Aβ_25−35_ can bind two ERp57 sites, that is a high‐affinity site and a low‐affinity site. Our SPR experiment does not indicate which one of the ERp57 domains is involved in the binding of Aβ_25−35_, so we decided to perform an in silico docking in order to elucidate the putative sites of interaction.

### 3.4 In silico docking demonstrates two main sites of interaction between ERp57 and Aβ_25−35_

**FIGURE 4:**
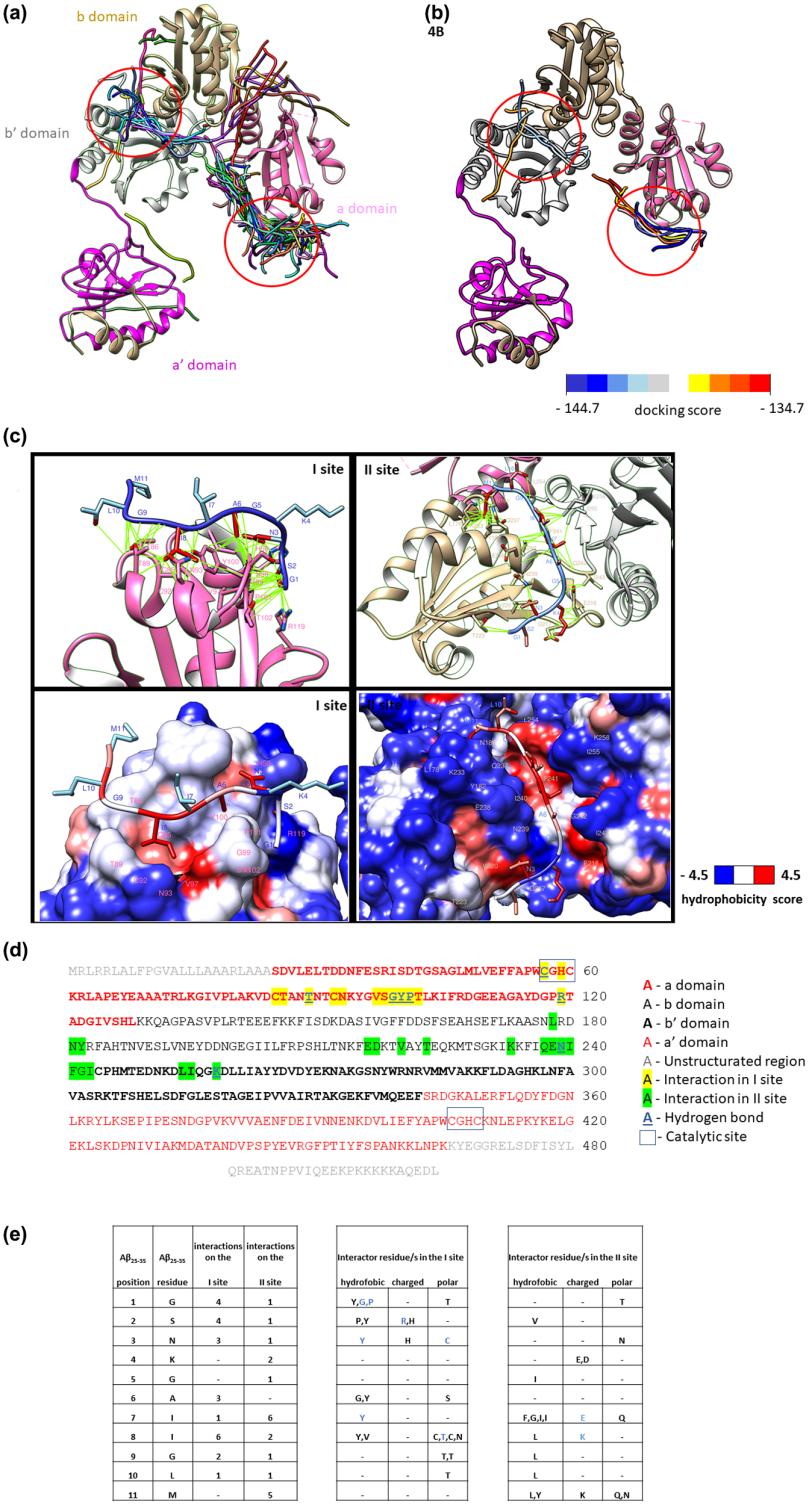
ERp57 protein structure: (a) result of the docking of Aβ_25−35_. The best 100 poses of the peptide are represented. (b) according to the energy score (ITScore) calculated by the HPEPDOCK server, the top 10 poses of Aβ_25−35_ are shown. The RMSD value between the 10 poses is 0.892 Ångström. (c) Top panel: detailed representation of the best poses in the two putative sites of interaction; interactions are colored in green. Bottom panel: representation of the protein surface according to the hydrophobic score (blue for hydrophilic region, red for hydrophobic region) (d) ERp57 sequence, colored according to domains and interactor residues. (e) schematic representation of the interaction. On the left, the sequence of Aβ_25−35_ and the number of engaged interactions. On the right, the protein residues that have engaged in the interactions with the Aβ_25−35_ peptide. The residues that form hydrogen bonds with the peptide are in light blue.

The docking of the Aβ_25−35_ fragment (pdb: 1QXC) was performed through the HPEPDOCK server (HPEPDOCK Server (hust.edu.cn]) (Zhou et al., 2018). The best 100 predicted poses, shown in Figure 4a, highlight two highly populated regions at the level of the a and b protein domains. We also took into account the top 10 poses (Figure 4b).

As representants, we choose two poses, one for each site, based on the top score. The two poses (Figure 4c, top images) show that the peptide establishes two very similar types of interaction with ERp57 protein. An overall assessment shows that both conformations engaged interaction with 46% of hydrophobic residue and the remained percentage is engaged by charged/polar residues of the protein. The first pocket possesses a more hydrophilic region (H59, R119, T102) that allows binding to the amino‐terminal residues of the peptide (G1, S2, N3), stabilized by 3 hydrogen bonds and the presence of Y100 that interacts with all three residues, an important region of low hydrophilicity characterized by A6 that establishes very important interactions with S98, G99, Y100, and a region of high hydrophobicity (Y100, V97) that favors interaction with the most hydrophobic residues of the molecule (I7,I8). Y100 appears to be a very important piece of docking as it interacts with six of the 11 residues of the peptide (G1, S2, N3, A6, I7, I8). Moreover, the peptide interacts with one of the two cysteines in the disulfide catalytic domain (Figure 4d, in the blue box).

The second pocket is located between the b and bʹ domains of the protein. Again, the amino terminal amino acids of the peptide (G1, S2, N3, K4) interact with a hydrophilic region where residues E216, D217, N239, V220, and T223 are present; residues G5 and A6 are located in a slightly hydrophobic region, where residue G5 interacts with residue I243, while the carboxy‐terminal region of the peptide is located in a highly hydrophobic region, which then stabilizes the hydrophobic residues of the peptide, in part because of the presence of two hydrogen bonds. Finally, methionine‐11 engages five interactions with L178, N181, Y182, K233, and G237. Also present here is a hydrophobic residue, L254, capable of interacting with three of the 11 peptide residues (I8, G9, L10) accompanied by I255 binding I7.

### 3.5 ERp57 decreases aggregation of Aβ_25−35_ in vitro

**FIGURE 5:**
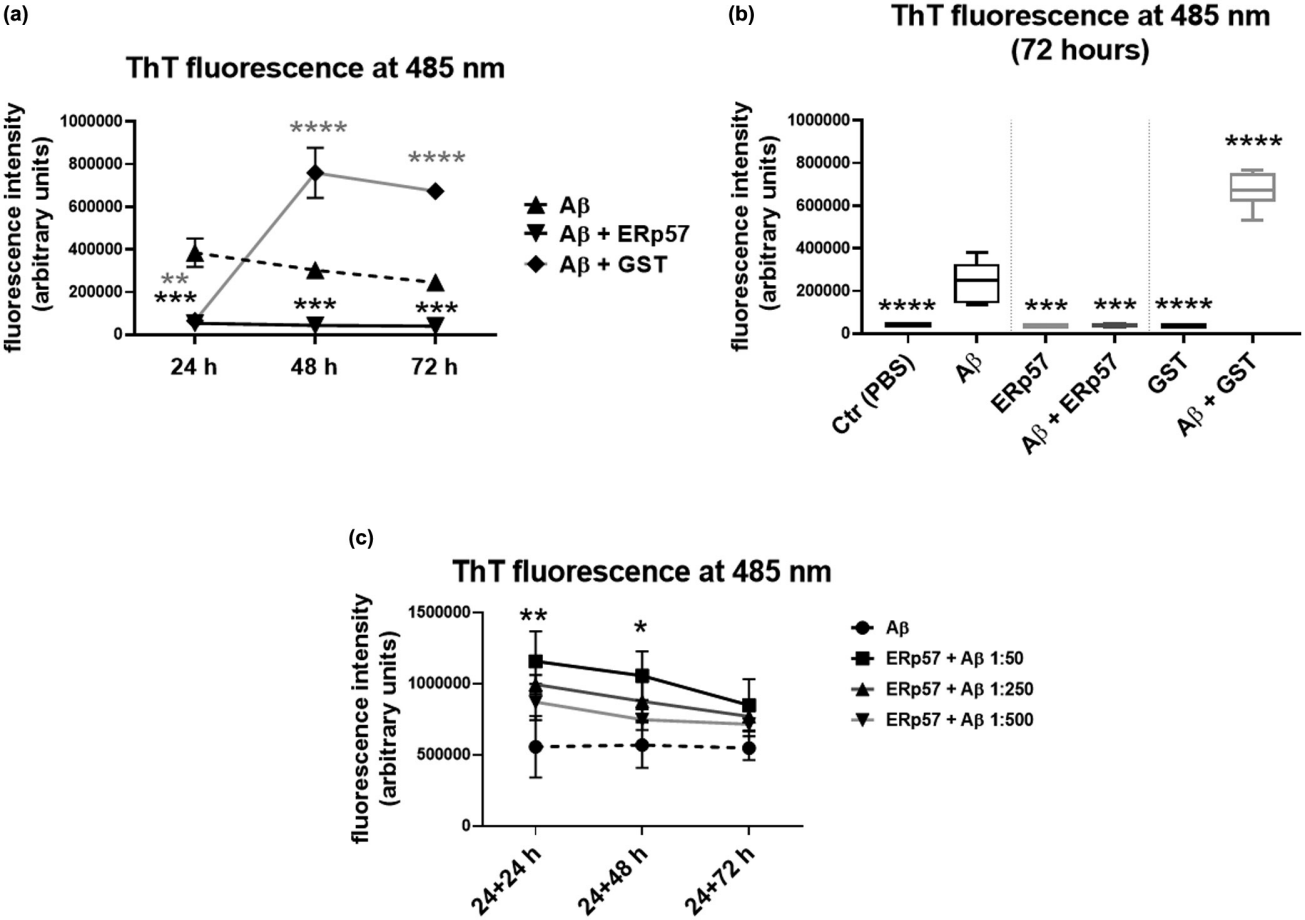
ERp57 reduces β‐amyloid_25−35_ aggregation after 72 h of incubation: (a) kinetic representation of the aggregation process of Aβ_25−35_ alone or in the presence of ERp57 or GST‐purified proteins (𝑛 = 6, number of independent Aβ_25−35_ incubations); data are represented as mean ± SD; (b) box plots of the 72 h timepoint; data are represented as box plots: lines represent the median and the whiskers represent min and max values; (c) kinetics representation of the aggregation process of Aβ_25−35_ alone or in the presence of different concentration of ERp57 after 24 h of Aβ_25−35_ aggregation (𝑛 = 3, number of independent Aβ_25−35_ incubations); data are represented as mean ± SD. Statistical comparison was performed using one‐way ANOVA. ****p < 0.0001 versus Aβ_25−35_.

Following demonstration of in vitro interaction, we used the thioflavin‐T, able to bind beta‐sheet structures, to measure the effects of ERp57 on Aβ_25−35_ aggregation. Particularly, when Aβ_25−35_ was aggregated in vitro for 72 h and incubated in the presence of thioflavin T, a fluorescence signal was observed with a maximum emission at 485 nm after excitation at 440 nm. The intensity of the signal was proportional to the extent of aggregation (Figure 5a).

Conversely, when Aβ_25−35_ was aggregated in the presence of recombinant ERp57 protein, a marked reduction in the fluorescence signal was observed, which indicates a lower level of aggregation of Aβ_25−35_. In fact, as shown in Figure 5a, the fluorescence intensity of aggregated Aβ_25−35_ alone and of Aβ_25−35_ aggregated in the presence of ERp57 are clearly separated, and there is a significant decrease in absorption at 485 nm in the Aβ_25−35_ + ERp57 combination compared to the signal intensity recorded for Aβ_25−35_ alone (Figure 5b). Therefore, ERp57 is not only able to interact with Aβ_25−35_ but is also capable to inhibit the aggregation of Aβ_25−35_, even in a 1:250 ERp57/Aβ_25−35_ molar ratio.

In order to verify the specificity of ERp57 to hamper Aβ_25−35_ aggregation, we used Glutathione‐S‐Transferase (GST) as a control protein. As shown in Figure 5a, GST showed a biphasic behavior. It was able to hamper Aβ_25−35_ aggregation within the first 24 h, however, in prolonged incubations a marked increase in fluorescence was observed compared to Aβ_25−35_ alone or Aβ_25−35_ aggregated in the presence of ERp57. It can be hypothesized that GST protein is able to slow down the process of Aβ_25−35_ aggregation but not to hamper it, contrary to ERp57 which completely blocks this process (Figure 5). These data suggest that ERp57 avoids the aggregation of the Aβ_25−35_ peptide in vitro, as demonstrated by Thioflavin‐T experiments.

Having ascertained the ability of ERp57 to block the aggregation process, we wondered if the protein was able to reverse a preformed amyloid fibril into oligomeric/monomeric forms. To understand this, we aggregated Aβ_25−35_ alone for 24 h and then incubated it for 72 h in the presence of increasing concentrations of ERp57, assessing every 24 h the state of aggregation by thioflavin T assay (Figure 5c). To our surprise, ERp57 was not able to reverse the amyloid aggregation. Moreover, we saw that the fluorescence intensity increased in a dose‐dependent manner with the presence of ERp57. Probably this phenomenon could be because of the fact that the presence of a protein capable of interacting with Aβ_25−35_ (given the previous experiments) can favor to the maintenance of Aβ_25−35_ in solution, facilitating the process of aggregation. Otherwise, if the ERp57 interacts with non‐aggregated Aβ_25−35_, the protein immediately binds the peptides and prevents them from reaching advanced states of aggregation.

### 3.6 ERp57 rescues the reduction in SH‐SY5Y cellular viability induced by Aβ_25−35_

**FIGURE 6:**
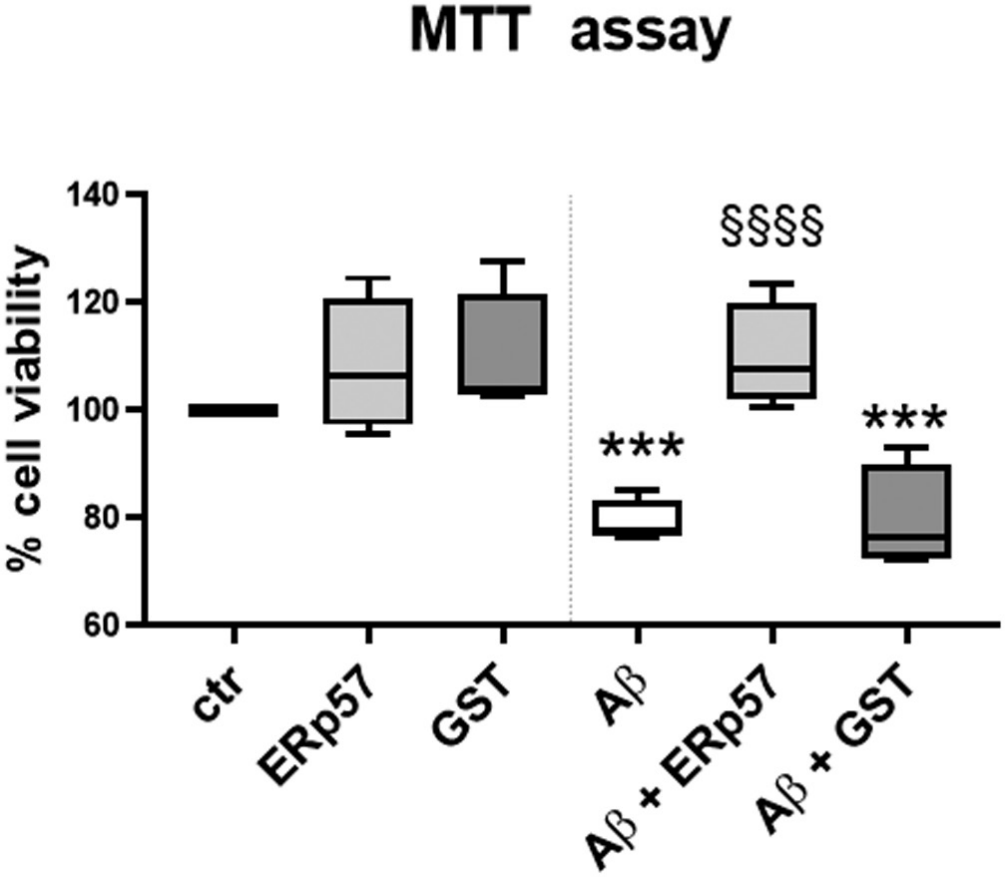
Protective effect of ERp57 on SH‐SY5Y cells treated with A𝛽_25−35_. SH‐SY5Y cells were incubated for 24 h with 30 M A𝜇_25−35_ that was aggregated for 72 h either alone or in the presence of ERp57 or GST recombinant protein in a 1:250 ratio. Cell viability was determined by using the MTT reduction assay and expressed as percentage compared to control Data are represented as box plot, lines represent the median and the whiskers represent min and max values (𝑛 = 4), 𝑛 = number of independent cell culture preparations, ***p < 0.001 versus ctr; ^§§§§^ *p* < 0.0001 versus A𝛽_25−35_. Statistical comparison was performed using two‐way ANOVA.

To expand our findings, we aimed to investigate whether recombinant ERp57 protein might protect cultured cells from Aβ_25−35_‐induced toxicity. We treated SH‐SY5Y neuronal cells with 30 μM Aβ_25−35_ aggregated for 72 h alone or in the presence of purified recombinant ERp57 or GST at a final concentration of 0.12 μM (1:250 molar ratio) and analyzed cell viability through MTT assay. The results presented in Figure 6 show a significant increase in cell viability after 24 h of treatment with a combination of ERp57 and Aβ_25−35_, compared to treatment with Aβ_25−35_ alone and Aβ_25−35_ with GST, whereas ERp57 and GST alone have no effect on cell viability.

This indicates that the co‐incubation of Aβ_25−35_ with recombinant ERp57 negatively affects the capacity of Aβ_25−35_ to induce cytotoxicity. We, therefore, show that the ability of recombinant ERp57 to block aggregation of Aβ_25−35_ and the consequent fibrils formation, has a direct, specific protective effect on cellular viability. As expected, since GST is not able to hamper Aβ_25−35_ aggregation, incubation of Aβ_25−35_ with GST does not exert protection against cytotoxicity. These data provide direct evidence on ERp57 ability to counteract Aβ_25−35_ toxicity in cellular model.

## 4 DISCUSSION

### 4.1 ERp57, inside and outside the cell

ERp57 is an endoplasmic reticulum disulfide isomerase which modulates folding of newly synthesized glycoproteins by recognizing and interacting with peptides or incorrectly folded proteins, thereby promoting formation of disulfide bonds and correct protein folding (Torres et al., 2015). Conversely, Aβ is a small peptide that, because of its intrinsic characteristics, tends to aggregate and to escape the ER‐associated degradation process (ERAD) (Holtzman, 2013). Eventually, monomeric Aβ is released out of the cell and begins its slow aggregation into fibrils that will form the template for subsequent oligomers (Törnquist et al., 2020). The present work was undertaken to test the hypothesis that ERp57 is released outside the neuronal cell following stress induced by amyloid aggregation, thus binding Aβ and blocking its toxic effects. This hypothesis stems from the first studies by Erickson (Erickson et al., 2005), who found ERp57 linked to Aβ in the cerebrospinal fluid of healthy people, suggesting that it may be a carrier protein which prevents Aβ aggregation.

When SH‐SY5Y neuroblastoma cells were treated with Aβ_25−35_ the intracellular level of ERp57 was found to be reduced within 1 h of treatment and this decrease was paralleled by an increase of the same protein in the culture medium in the same timeframe. After 24 h, the level of the protein returned to basal levels both intracellularly and extracellularly. Notably, the extracellular presence of ERp57 was observed also in untreated cells. Further evidence of a rearrangement of ERp57 protein in treated cells was provided by the immunofluorescence images that revealed a dramatic change in ERp57 signal in the cell periphery. After 3 h of treatment with Aβ_25−35_ we observed how the fluorescence is homogeneously diffused throughout the cell, suggesting an early rearrangement of ERp57 protein; on the contrary, after 6 h, ERp57 was mainly displaced in the periphery of the cell, probably to guarantee the main function of ERp57, that is the correct folding of the glycosylated proteins at the level of the synapses. 24 h after treatment, the cells reacquired the original cellular distribution of ERp57. These data support our hypothesis that ERp57 is released within the first hours of treatment and is then subsequently re‐distributed within the cell.

### 4.2 ERp57‐Aβ interaction and biological significance

Many literature reports indicate that ERp57 is a multifunctional protein with a high propensity to interact with peptides and proteins prone to misfolding, such as PrP (Hetz et al., 2005; Thapa et al., 2018; Torres et al., 2015) and α‐synuclein (Serrano et al., 2020). Moreover, other members of the PDI family are able to bind tau protein (Xu et al., 2013) and proteins without disulfide bonds (Cai et al., 1994). Additionally, Irvine has demonstrated that PDI binds more effectively the unfolded proteins than the folded ones (Irvine et al., 2014). As ERp57 belongs to the PDI family, this could imply that it is able to recognize specific structural features responsible for the aggregation process. Here, we demonstrated that ERp57 binds Aβ_25−35_ with micromolar affinity, and that ERp57 impairs Aβ_25−35_ peptide aggregation. SPR experiments indicated that ERp57 is able to bind to Aβ_25−35_ peptide, which is a short fragment of the full‐length Aβ_1−42_. The dissociation phase was found to be biphasic, an indication that Aβ_25−35_ can bind two ERp57 sites, that is a high‐affinity site and a low‐affinity site.

Our in silico study reinforced these data and showed that the first pocket is placed on the domain, in the proximity of the CHGC catalytic site, in line with the description of Xu and coworkers (Xu et al., 2013), who demonstrated that PDI protein is able to reduce the formation of tau fibrils within the cell, by interacting with the thioredoxin‐like catalytic domain a and aʹ; on the other hand, the second pocket is between the bʹ and b domains, in line with Kozlov, who proposed that the bʹ domain of ERp57 provides the majority of the binding site with substrates, while the b domain affords additional contacts (Kozlov et al., 2006), strengthening the interaction.

The thioflavin T experiments demonstrated that the interaction between Aβ_25−35_ and ERp57 strongly reduced the aggregation of the peptide but was not able to disassemble pre‐aggregated Aβ_25−35_. We thus hypothesize that the mechanism by which ERp57 exerts its neuroprotective action resides in ERp57 impairment of peptide aggregation, rather than in disaggregation of existing fibrils. The inhibition of the aggregation was induced by low amounts of ERp57 protein, present in the reaction mixture in a very low molar ratio compared to the peptide (1:250), in line with the experiments conducted by Serrano and coworkers, who demonstrated that ERp57 is able to reduce the aggregation of α‐synuclein in a very efficient manner, in a 1:50 molar ratio (Serrano et al., 2020). The efficiency of ERp57, even at a low molar ratio compared to Aβ_25−35_, indicates that ERp57 may bind oligomeric Aβ_25−35_.

We also investigated the effects of cell treatment with Aβ_25−35_ aggregated in the presence or in the absence of ERp57. The experiments demonstrated that ERp57 has a direct protective effect on cellular viability, as a consequence of aggregation inhibition. Indeed, when SH‐SY5Y cells were treated with Aβ_25−35_ in the presence of ERp57, their viability was preserved. Based on this evidence, it can be hypothesized that following Aβ_25−35_ stress the cell senses the presence of misfolded proteins and releases ERp57 in an attempt to dampen amyloid toxicity. Once ERp57 is outside the cell, it is able to bind Aβ, thus inhibiting the aggregation process. We can speculate that the ERp57‐Aβ_25−35_ complex could be then subjected to phagocytosis and digested by the glia, but, in any case, the complex is not harmful for the cells, as demonstrated in viability assay or in vivo (Erickson et al., 2005).

### 4.3 ERp57 in neurodegenerative diseases

As previously reported (Williams, 2006), it is known that ERp57 together with calreticulin and calnexin forms a complex capable of interacting with glycoproteins, including APP, at the Golgi level, and therefore it can be hypothesized that the contact regions between ERp57 and APP are maintained at least in part also by Aβ, despite the cuts suffered by the secretases. Indeed, Selivanova (Selivanova et al., 2007) has demonstrated that ERp57 in permeabilized cells interacts with full‐length APP during the process of O‐glycosylation, whereas it does not interact with the c99 fragment of APP generated by the β‐secretase activity; nevertheless, the same author admits that the interaction observed by Erickson (Erickson et al., 2005) could be explained by a different interaction between ERp57 and Aβ_1−42_.

Holtzman hypothesizes that a decline in the ER ability to catalyze post‐translational changes on the protein APP could be at the base of AD. Indeed, the N‐glycosylation of the APP is a fundamental step for the subsequent cleavage by the secretases and the folding assisted by the calreticulin‐ERp57 complex. If the APP does not undergo the correct post‐translational changes and is not folded correctly, the resulting Aβ peptides aggregate rapidly and become too bulky to undergo the process of ER‐associated degradation (ERAD). Thus, aggregated Aβ remains in the lumen of the ER and is eventually secreted outside the cell (Erickson et al., 2005; Holtzman, 2013).

In line with this theory, Chun demonstrated that the correct glycosylation of the residue T576 of the APP is fundamental for the correct cleavage of the APP (Chun, Kwon, et al., 2015). Correct glycosylation of APP decreases its endocytosis and increases trafficking from Trans‐Golgi‐Network to the cell surface, resulting in an increase in non‐amyloidogenic processing and a decrease in Aβ production (Chun, Park, et al., 2015).

ERp57 is able to guarantee the correct folding of APP after glycosylation, but it could also be able to perform a regulatory function outside the cell, binding Aβ and possibly facilitating its phagocytosis and digestion by the microglial cells.

In this context, our work shows how ERp57 is able to interact with Aβ_25−35_ probably by two different sites, with micro‐molar affinity. In vitro experiments show that ERp57 is able to bind Aβ _25−35_ probably in an oligomeric form (considering the sub‐stoichiometric ratio of the assay), but fails to disaggregate a fibril, while cell experiments show that Aβ_25−35_ bound by ERp57 no longer leads to toxicity.

It could be speculated that in AD patients this regulation is lost, possibly because less ERp57 is secreted or the oxidative environment is not favorable, because of the aging process (Ghosh & Brewer, 2014) and therefore the Aβ, released outside the cell, is no longer digested by enzymes such as neprilysin (Campos et al., 2020) and Insulin Degrading Enzyme (Bulloj et al., 2010), triggering a fibrillation process that will eventually lead to the formation of amyloid aggregates (Erickson et al., 2005; Zhao et al., 2012). To date, it was reported that neuronal (Fontana et al., 2020) and SH‐SY5Y cells are able to internalize Aβ (Ida et al., 1996) and pre‐formed alpha‐synuclein fibrils (Pantazopoulou et al., 2021) through endocytosis and clear them, so it could be hypothesized that ERp57, which is able to bind alpha‐synuclein (Serrano et al., 2020), is released from the neuronal cells in order to bind the Aβ, prevent its aggregation and favor the endocytosis of this complex.

### 4.4 Future perspectives

To date, there are no cures for AD, and the currently approved drugs can only mitigate symptoms such as depression and agitation (Arvanitakis et al., 2019). Hence, Aβ production and clearance are key pathways in the development of therapeutic strategies for AD. Finding protein interactors able to bind Aβ and reduce its aggregation could represent a possible therapeutic approach to slow down the onset and progression of the disease, especially in cases of familial AD (Magzoub, 2020; Soares et al., 2021). ERp57 has been studied extensively in the literature, since it is an important chaperone, but only recently attention was paid to the functions that it can perform in the extracellular space in relation to protein aggregates. Our work provides clues on the possible role of ERp57 in AD pathogenesis and suggests recombinant ERp57 peptides as a possible therapeutic approach for this pathology. Interestingly, new therapeutic approaches focus on recombinant proteins which interact with Aβ hampering its aggregation (Magzoub, 2020), hence ERp57 could represent a possible therapeutic tool useful in counteracting neurodegeneration induced by amyloid aggregation.

#### AUTHOR CONTRIBUTION

F.A, L.M., and G.C. conceived the project. D.D.R., D.R., I.M., F.G., M.G., G.C. L.M., and P.M. performed the experiments. A.F. and A.V.V. synthesized the Aβ_25−35_. All authors analyzed and discussed the data. L.M., G.C., F.G., and D.D.R. drafted the manuscript. The manuscript was written through contributions of all authors, and all authors have given approval to the final version of the manuscript.

#### CONFLICT OF INTEREST

The authors declare no competing financial interest.

## Supporting information


Data S1

## Data Availability

The data that support the findings of this study are available from the corresponding author upon reasonable request.
