# Supplementary material for: ERp57 chaperon protein protects neuronal cells from Aβ‐induced toxicity
Source: J Neurochem. 2022 Jul 8;162(4):322–36. doi: 10.1111/jnc.15655 (PMC9543391; doi:10.1111/jnc.15655)
Supplement: Supplementary file 1 — Data S1 [file JNC-162-322-s001.pdf]

ERp57 Chaperon Protein Protects Neuronal Cells from Aβ-induced toxicity

Daniel Di Risola[1§], Daniela Ricci[1,2§], Ilaria Marrocco[1,3], Flavia Giamogante[1,4], Maddalena Grieco[1], Antonio Francioso[1], Aldrin Vasco-Vidal[4], Patrizia Mancini[5], Gianni Colotti[6*], Luciana Mosca[1*], Fabio Altieri[1]

[1] Department of Biochemical Sciences, Sapienza University of Roma, Rome, Italy
[2] Present address: Immunobiology of Infection Unit, Institut Pasteur, Paris, France
[3] Present address: Department of Biological Regulation, Weizmann Institute of Science, Rehovot, Israel
[4] Present address: Institute of Oncology Research (IOR), Bellinzona, Switzerland
[4] Leibniz Institute of Plant Biochemistry. Weinberg, 3. 06120 Halle (Saale), Germany
[5] Dept Experimental Medicine, Sapienza University of Roma, Rome, Italy
[6] Institute of Molecular Biology and Pathology – Italian National Research Council, Rome, Italy

§ co-first authors
* corresponding authors

Statistical table, referring to the image 1B

	ctr	1 h	3 h	6 h	24 h
Number of values	4	4	4	4	3*
**T-test**					
degree of freedom (df)		6	6	6	5
F-value		infinity	infinity	infinity	infinity
p-value		0,025	0,1458	0,4573	0,079
Mean	100	70,76	114,7	95,42	134,2
Std. Deviation	0	19,16	17,61	11,54	32,21
Std. Error of Mean	0	9,582	8,807	5,768	18,59

*one sample (24 h) was lost

Statistical table, referring to the image 1D

	ctr	1 h	3 h	6 h	24 h
Number of values	8	7*	7*	8	7**
**T-test**					
degree of freedom (df)		13	13	14	13
F-value		infinity	infinity	infinity	infinity
p-value		<0,0001	<0,0001	0,7138	0,0548
Mean	100	135,5	132	97,09	125,4
Std. Deviation	0	18,55	16,08	21,99	34,29
Std. Error of Mean	0	7,01	6,077	7,775	12,96

*two samples (1-3h) were excluded because exceeded ±2 SD

**one sample (24 h) was lost

Statistical table, referring to the image 5A

	24 h	48 h	72 h
Number of values	6	6	6
**1way ANOVA**			
degree of freedom (df)	5	5	5
F-value	9,422	115,2	133,4
Adjusted P Value	<0,0001	<0,0001	<0,0001

**Tukey's multiple comparisons test**	**Adjusted P Value**	**Adjusted P Value**	**Adjusted P Value**
Ctr (PBS) vs. ERp57	>0,9999	>0,9999	>0,9999
Ctr (PBS) vs. GST	>0,9999	>0,9999	>0,9999
Ctr (PBS) vs. Aβ	<0,0001	<0,0001	<0,0001
Ctr (PBS) vs. Aβ+ ERp57	0,9998	>0,9999	>0,9999
Ctr (PBS) vs. Aβ+ GST	0,9974	<0,0001	<0,0001
ERp57 vs. GST	>0,9999	>0,9999	>0,9999
ERp57 vs. Aβ	<0,0001	<0,0001	<0,0001
ERp57 vs. Aβ+ ERp57	0,9997	>0,9999	>0,9999
ERp57 vs. Aβ+ GST	0,9966	<0,0001	<0,0001
GST vs. Aβ	0,0001	<0,0001	<0,0001
GST vs. Aβ+ ERp57	>0,9999	>0,9999	>0,9999
GST vs. Aβ+ GST	0,9985	<0,0001	<0,0001
Aβ vs. Aβ+ ERp57	0,0002	<0,0001	<0,0001
Aβ vs. Aβ+ GST	0,0003	<0,0001	<0,0001
Aβ+ ERp57 vs. Aβ+ GST	>0,9999	<0,0001	<0,0001

Statistical table, referring to the image 5B

	**Ctr (PBS)**	**ERp57**	**GST**	**Aβ**	**Aβ+ ERp57**	**Aβ+ GST**
Number of values	6	6	6	6	6	6
Mean	41333	34174	36926	244398	39396	673123
Std. Deviation	975,5	1415	1575	101078	5359	86318
Std. Error of Mean	398,2	577,8	643,1	41265	2188	35239

Statistical table, referring to the image 5C

	24+24h	24+48h	24+72h
Number of values	3	3	3

**1way ANOVA**			
degree of freedom (df)	3	3	3
F-value	6,893	6,41	2,471
Adjusted P Value	0,0131	0,016	0,1363

**Tukey's multiple comparisons test**	**Adjusted P Value**	**Adjusted P Value**	**Adjusted P Value**
Aβ vs. ERp57 + Aβ 1:50	0,01	0,0121	0,1132
Aβ vs. ERp57 + Aβ 1:250	0,0501	0,1051	0,2851
Aβ vs. ERp57 + Aβ 1:500	0,1774	0,4514	0,5007
ERp57 + Aβ1:50 vs. ERp57 + Aβ1:250	0,6513	0,4475	0,9009
ERp57 + Aβ1:50 vs. Aβ+ ERp57 1:500	0,2353	0,104	0,6646
ERp57 + Aβ1:250 vs. Aβ+ ERp57 1:500	0,8044	0,6887	0,9614

24+24h	Aβ	ERp57 + Aβ1:50	ERp57 + Aβ1:250	Aβ+ ERp57 1:500
Number of values	3	3	3	3
Mean	556239	1156681	994315	870873
Std. Deviation	216544	211130	66909	128277
Std. Error of Mean	125022	121896	38630	74061

Statistical table, referring to the image 6

	ctr	Aβ	ERp57	Aβ + ERp57	GST	Aβ + GST
Number of values	4	4	4	4	4	4
**2way ANOVA (column factor)**						
degree of freedom (df)	5					
p-value	<0,0001					
Adjusted P Value		0,0005	<0,0001	<0,0001	<0,0001	>0,9999
Mean	100	79,14	108,3	109,9	109,6	79,61
Std. Deviation	0	3,99	12,43	9,826	11,99	9,695
Std. Error of Mean	0	1,995	6,215	4,913	5,995	4,847
